# Clinical Outcomes and Prognostic Factors of Kaposi Sarcoma in a Non-Endemic Region: A Retrospective Cohort Study

**DOI:** 10.3390/cancers18213559

**Published:** 2026-11-05

**Authors:** Omar Hamdy, Eman Eid, Rana Reda, Ahmad E. Eladl, Mariam Harby, Yasmeen Osama, Esraa Abdelkhalek, Sara Genedi, Ibrahim Saleh Alawadi, Bassant Atef Ahmed, Mohamed Elbadrway

**Affiliations:** 1 Surgical Oncology Department, Oncology Center, Mansoura University, Mansoura 35516, Egypt; m_s2014@mans.edu.eg; 2 Medical Oncology Unit, Oncology Center, Mansoura University, Mansoura 35516, Egypt; emaneid@mans.edu.eg (E.E.); ranamohamed94@mans.edu.eg (R.R.); 3 Pathology Department, Faculty of Medicine, Mansoura University, Mansoura 35516, Egypt; ah_eladl@mans.edu.eg; 4 Mansoura University Hospitals, Mansoura 35516, Egypt; mariamnabil256@std.mans.edu.eg (M.H.); yasminosama42@std.mans.edu.eg (Y.O.); esraaabdelkhalek@std.mans.edu.eg (E.A.); saragenedi@std.mans.edu.eg (S.G.); 5 Mansoura Manchester Program for Medical Education, Faculty of Medicine, Mansoura University, Mansoura 35516, Egypt; ebraheem44444@std.mans.edu.eg; 6 Faculty of Medicine, Tanta University, Tanta 31527, Egypt; ug_31186045@med.tanta.edu.eg

**Keywords:** kaposi sarcoma, HIV, prognosis, survival

## Abstract

**Background:** Kaposi sarcoma (KS) is an unusual disease that presents in various clinical forms, leading to variable treatment plans. In this study, we present our center’s experience with the diagnosis and management of KS in a non-endemic area, aiming to highlight the distinctive characteristics of this disease in our locality. **Methods:** This is a retrospective study that included all patients with KS who presented to our center between January 2008 and December 2025. The epidemiological, clinical, therapeutic, and prognostic data were analyzed. Given the limited number of events, all survival and regression analyses were exploratory and hypothesis-generating. **Results:** A total of 56 patients with KS (predominantly male: 46/56, 82.1%) were included in the study. The median age at diagnosis was 61 years. HIV seropositivity was documented in 13 patients (23.6%), HCV in 11 (21.2%), and HBV in one patient (1.9%). Nodular morphology was the most common presentation, followed by plaque-type and patch-stage. More than 50 lesions were observed at presentation in 25 patients. The median largest tumor diameter was 2.0 cm (range: 0.5–30.0 cm). Systemic chemotherapy was administered to 29 patients (51.8%), representing the most common initial approach. Surgical excision alone was performed in 13 patients (23.2%), radiotherapy alone in 3 (5.4%), cryotherapy alone in 2 (3.6%), and intralesional chemotherapy in 2 (3.6%). Local recurrence occurred in 12.5% of patients, with the median time to recurrence of 17 months, and positive lymph node status at diagnosis was the only variable associated with local recurrence in exploratory univariate analysis. Metastatic disease developed in 16.1% of patients, with a median time to metastasis of 10 months, and nodular lesion morphology and Ki-67 proliferation index were associated with metastasis in exploratory multivariate analysis. At a median follow-up of 40 months, 9 deaths (16.1%) were recorded. HIV-positive status was associated with worse overall survival (OS). These findings require validation in larger, adequately powered cohorts. **Conclusions:** While KS generally shows an indolent behavior, exploratory analysis suggests that nodular morphology, high Ki-67, lymph node involvement, and HIV seropositivity may be associated with worse outcomes. Systemic therapy remains the most frequently used treatment for advanced disease, while surgery and radiotherapy are appropriate options for selected localized lesions. These findings should be considered hypothesis-generating and require confirmation in larger, prospective, multicenter studies.

## Simple Summary

Kaposi sarcoma is a rare cancer that can appear in different forms and may require different treatment approaches. In this study, we reviewed patients with Kaposi sarcoma treated at our center over an 18-year period in a non-endemic area to better understand how the disease presents and behaves in our population. We found that most patients were older men, and about one-quarter were living with HIV infection. The disease often presented with multiple lesions, and systemic treatment was the most used approach, particularly in patients with more advanced disease. Although Kaposi sarcoma was generally slow growing, patients with nodular lesions, lymph node involvement, or higher tumor cell activity may have a greater risk of progression. Patients with HIV infection also appeared to have poorer survival. These hypothesis-generating findings may help guide treatment decisions and improve understanding of Kaposi sarcoma in similar settings.

## 1. Introduction

Kaposi sarcoma (KS) is an unusual disease that affects different populations, presenting in various clinical forms with a wide range of severity. KS was first described by Moritz Kaposi in 1872 when he reported a case series of five patients based solely on clinical and dermatological observations, while its etiology remained unknown for decades. During the 1980s AIDS epidemic, the increased prevalence of the disease among immunocompromised patients led to the identification of human herpesvirus 8 (HHV-8) as the predisposing factor, more recently named Kaposi sarcoma-associated herpesvirus [1].

KS is a vascular tumor that affects the endothelium; its occurrence is influenced by immune status and geographic factors [2]. KS has four clinical subtypes: Classic type affects older men of Eastern European and Mediterranean descent on the lower limbs and is usually indolent; Endemic type affects children and adults in Sub-Saharan Africa, causing generalized lymphadenopathy and is usually aggressive; Epidemic type occurs in HIV patients and, while it causes multiple cutaneous lesions, can be highly aggressive without antiretroviral therapy; and Iatrogenic type affects organ transplant patients using immunosuppressive drugs, causing cutaneous lesions and affecting internal organs [1,3]. According to GLOBOCAN 2022, the overall global 5-year prevalence of KS is 82,122 cases, with Africa bearing the highest burden (67.4%) [3].

KS has various clinical forms, so the treatment plan also varies according to the case. The main treatment for AIDS patients who suffer from KS is combination antiretroviral therapy (cART) to control HIV replication and restore patient immunity; chemotherapy may be added according to the case severity. In patients who developed KS after organ transplantation, lowering the immunosuppressive dose has a beneficial role in treatment, but graft rejection may occur [4]. Using topical treatment only, such as timolol, imiquimod, and alitretinoin, is suggested for localized skin nodules [5]. HIV-related KS prognosis is dependent on the AIDS Clinical Trials Group (ACTG) staging system, which stratifies patients into good and poor risk based on tumor extent, immune status, and systemic illness [6,7].

In this study, we present the experience of a tertiary cancer referral center in the diagnosis and management of KS in a non-endemic area, aiming to highlight the main characteristics of such a unique disease in our locality.

## 2. Materials and Methods

Study design and setting: This retrospective study included all the patients with KS who presented to our center from January 2008 to December 2025. The epidemiological, clinical, therapeutic, and prognostic data were analyzed. IRB approval was obtained from the Institutional Review Board, Faculty of Medicine, Mansoura University. It has the number R.26.04.3683.R1.

### 2.1. Inclusion Criteria

Any patient with pathologically proven KS who underwent management during the study period at the center.

### 2.2. Exclusion Criteria

- Missing or unavailable data
- Unconfirmed diagnosis
- Concurrent malignancy
- Prior chemotherapy or radiotherapy

### 2.3. Variables

- Demographic: Age, sex, body mass index (BMI), and viral serostatus (HBsAg, HCVab and HIVab).
- Clinical: lesion morphology, number, diameter, biopsy, and type of surgery.
- Oncological: lymph node involvement, adjuvant treatment, recurrence, and survival.

Ki-67 was retrieved from pathological records and analyzed as a binary variable (≥15% vs. <15%) for consistency with prior literature. Continuous analysis and sensitivity analyses with alternative cutoffs were not performed due to the small number of patients with available Ki-67 data and the limited number of events. We acknowledge that this cutoff was not pre-specified in our study protocol and was chosen based on the distribution of our data, as the median ki-67 for the available data was 15%.

**Primary outcome**: The development of recurrence and disease-free survival.
**Secondary outcome:** The development of metastases and overall survival.
**Statistical Analysis:** Continuous variables were summarized as median (range, interquartile range) and categorical variables as frequencies (percentages), with missing data explicitly reported for all baseline and outcome variables. Overall survival (OS), disease-free survival (DFS), recurrence-free survival (RFS), and metastasis-free survival (MFS) were estimated using the Kaplan–Meier method, with survival distributions compared using the log-rank test. Univariate and multivariate Cox proportional hazards regression models were used to identify variables associated with local recurrence, metastasis, and OS. Multivariate models for metastasis were adjusted for age, sex, and HIV status; models for OS were adjusted for age, sex, HIV status, metastasis, and nodular morphology. The proportional hazards assumption was verified using Schoenfeld residuals (*p* > 0.05 for all covariates), indicating no significant violation. Complete-case analysis was performed without imputation of missing data. A two-sided *p*-value < 0.05 was considered statistically significant. All analyses were conducted using R version 4.2.1 (R Foundation for Statistical Computing, Vienna, Austria) with the survival and survminer packages for survival analyses and forest plot generation.

Given the retrospective nature of the study and the rarity of KS, missing data were unavoidable and may introduce selection bias. Missing data were handled using complete-case analysis without imputation. The extent of missing data is explicitly reported for all baseline and outcome variables in Tables. Competing risk analysis was not performed due to the small number of events and the exploratory nature of the study. Subgroup analyses were exploratory and not adjusted for multiple comparisons.

Multivariable model construction was guided by clinical relevance and the need to limit overfitting. For the metastasis model, we included age, sex, and HIV status as a priori confounders, plus nodular morphology and Ki-67 as variables of primary interest (5 covariates total; 9 events; events-per-variable [EPV] = 1.8). For the OS model, we included age, sex, HIV status, metastasis, and nodular morphology (5 covariates; 9 deaths; EPV = 1.8). Penalized regression methods (e.g., Firth’s penalized likelihood, ridge regression, LASSO) were not employed due to the exploratory nature of the study and the desire to present conventional hazard ratios. However, we acknowledge that the low EPV ratios substantially increase the risk of overfitting and unstable estimates.

Metastasis was included as a binary time-fixed covariate in the OS model, representing whether the patient ever developed metastatic disease during follow-up. We acknowledge that this approach is subject to immortal time bias. Time-dependent Cox regression or landmark analysis would be more appropriate but was not feasible due to the small number of events (*n* = 9 metastases, *n* = 9 deaths).

## 3. Results

### 3.1. Study Population and Baseline Characteristics

A total of 56 patients (predominantly males: 46/56, 82.1%) with KS were included in the study. The median age at diagnosis was 61 years (range: 27–89; interquartile range [IQR]: 43–73). HIV seropositivity was documented in 13 patients (23.6%), HCV in 11 (21.2%), and HBV in one patient (1.9%). The median body mass index (BMI) was 27.0 kg/m^2^. HIV status was available for 55 of 56 patients (1 missing). Among patients with available data: HIV-negative, *n* = 42 (76.4%); HIV-positive, *n* = 13 (23.6%). This classified our cohort into HIV-associated KS (*n* = 13, 23.6%) and Classic KS (*n* = 42, 76.4%). The baseline demographic and clinical characteristics are summarized in Table 1.

### 3.2. Tumor and Pathological Characteristics

Tumor characteristics are presented in Table 2. Nodular morphology was the most common presentation (28/56, 50.0% of patients with available data, *n* = 52), followed by plaque-type (15/56, 26.8%) and patch-stage (9/56, 16.1%). More than 50 lesions at presentation were observed in 25 patients. The median largest tumor diameter was 2.0 cm (range: 0.5–30.0 cm) among evaluable patients (*n* = 38).

Missing data were substantial for several variables: Ki-67 proliferation index was available for only 17/56 patients (30.4%; 39 missing), largest tumor diameter for 38/56 (67.9%; 18 missing), margin status for 10/13 surgically treated patients (76.9%), and lymph node status for 51/56 (91.1%; 5 missing). Immunohistochemical markers also had substantial missingness: HHV-8 (18/56 missing), CD34 (23/56 missing), and CD31 (34/56 missing).

**Figure 1:**
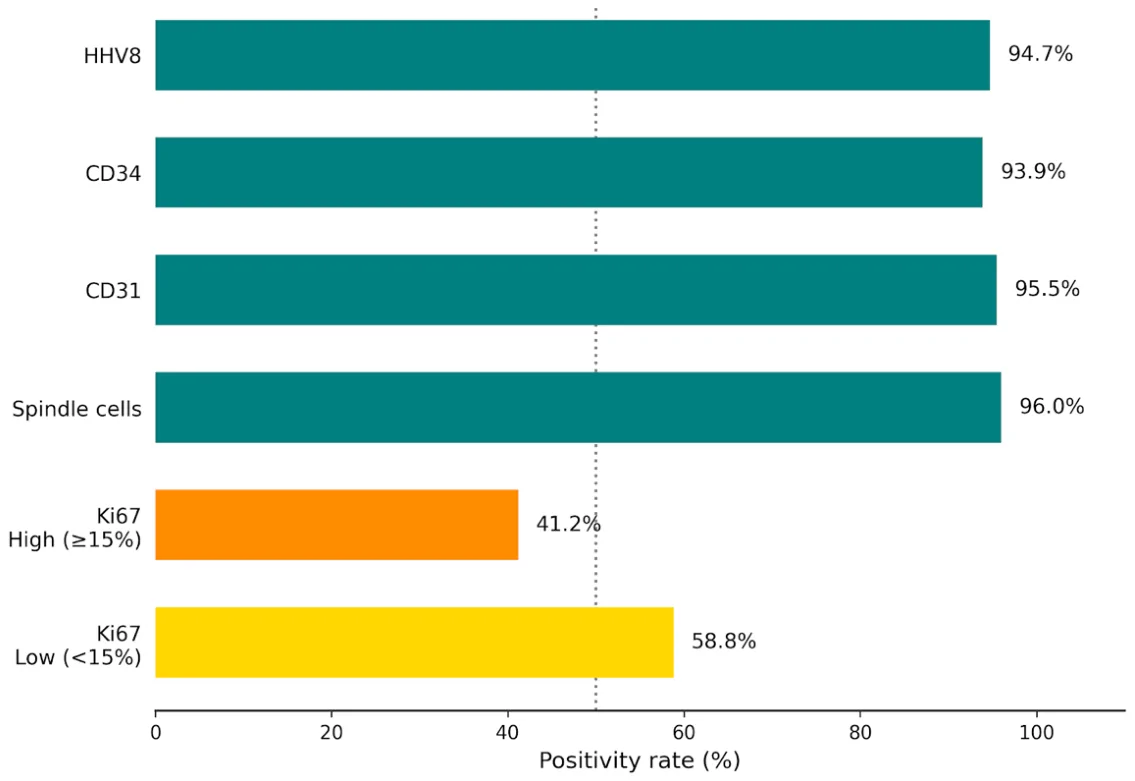
Immunohistochemical marker expression rates in KS lesions. Horizontal bar chart showing the percentage of evaluable samples with positive staining for HHV-8 (94.7%, *n* = 38), CD34 (93.9%, *n* = 33), and CD31 (95.5%, *n* = 22). Ki-67 (*n* = 17) is shown as high (≥15%) in 41.2% and low (<15%) in 58.8%. Note: No confidence intervals are displayed. Numbers above bars indicate the total number of evaluable samples per marker. See Table 2 for missing data.

Immunohistochemical analysis demonstrated high positivity rates for KS-associated markers (Figure 1): HHV-8 was positive in 36/38 (94.7%), CD34 in 31/33 (93.9%), and CD31 in 21/22 (95.5%). Spindle cells were identified in 48/50 evaluable patients (96.0%). The Ki-67 proliferation index, available for 17 patients, was classified as high (≥15%) in 7 (41.2%) and low (<15%) in 10 (58.8%).

### 3.3. Treatment Modalities

**Figure 2:**
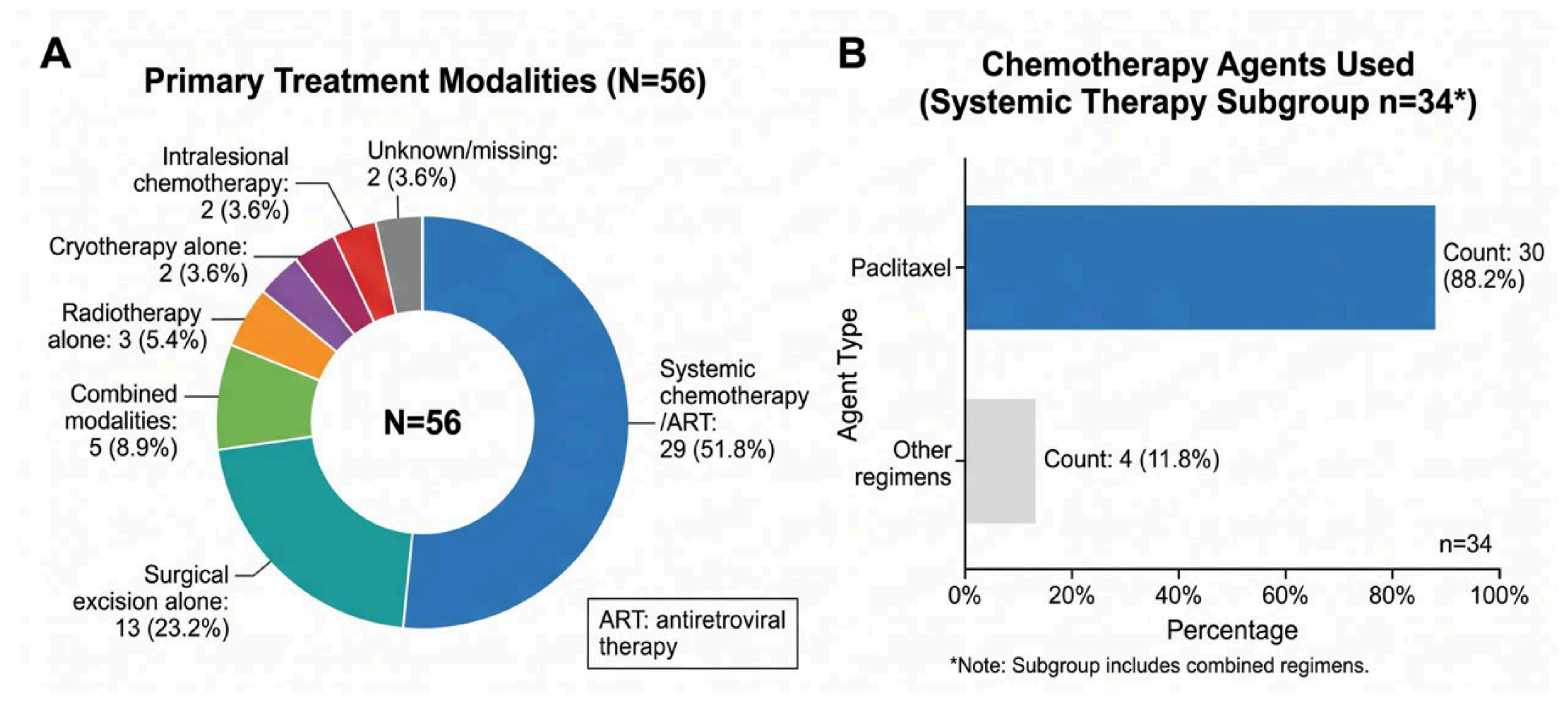
Treatment patterns in the cohort. (**A**) Primary treatment modalities among all 56 patients. Systemic chemotherapy/ART: 51.8%; surgery alone: 23.2%; combined modalities: 8.9%; radiotherapy alone: 5.4%; cryotherapy alone: 3.6%; intralesional chemotherapy: 3.6%; unknown/missing: 3.6%. (**B**) Chemotherapy agents among patients receiving systemic therapy (*n* = 34, including combined regimens). Paclitaxel: 88.2% (*n* = 30); other regimens: 11.8% (*n* = 4). See Table 3 for details.

Primary treatment distribution is detailed in Table 3 and visualized in Figure 2A. Systemic chemotherapy was administered as the primary initial treatment in 29 patients (51.8%), representing the most common initial approach. When including patients who received systemic therapy as part of combined-modality regimens (*n* = 5), a total of 34 patients (60.7%) received systemic therapy at some point during their treatment course. Surgical excision alone was performed in 13 patients (23.2%), radiotherapy alone in 3 (5.4%), cryotherapy alone in 2 (3.6%), and intralesional chemotherapy in 2 (3.6%). Combined modality therapy was utilized in 5 patients (8.9%).

Among the 34 patients who received systemic therapy (including combined regimens), paclitaxel was the predominant agent, used in 30 patients (88.2%) (Figure 2B). Among surgically treated patients with evaluable margin status (*n* = 10), negative (free) surgical margins were achieved in 7 (70.0%).

### 3.4. Recurrence Outcomes

**Figure 3:**
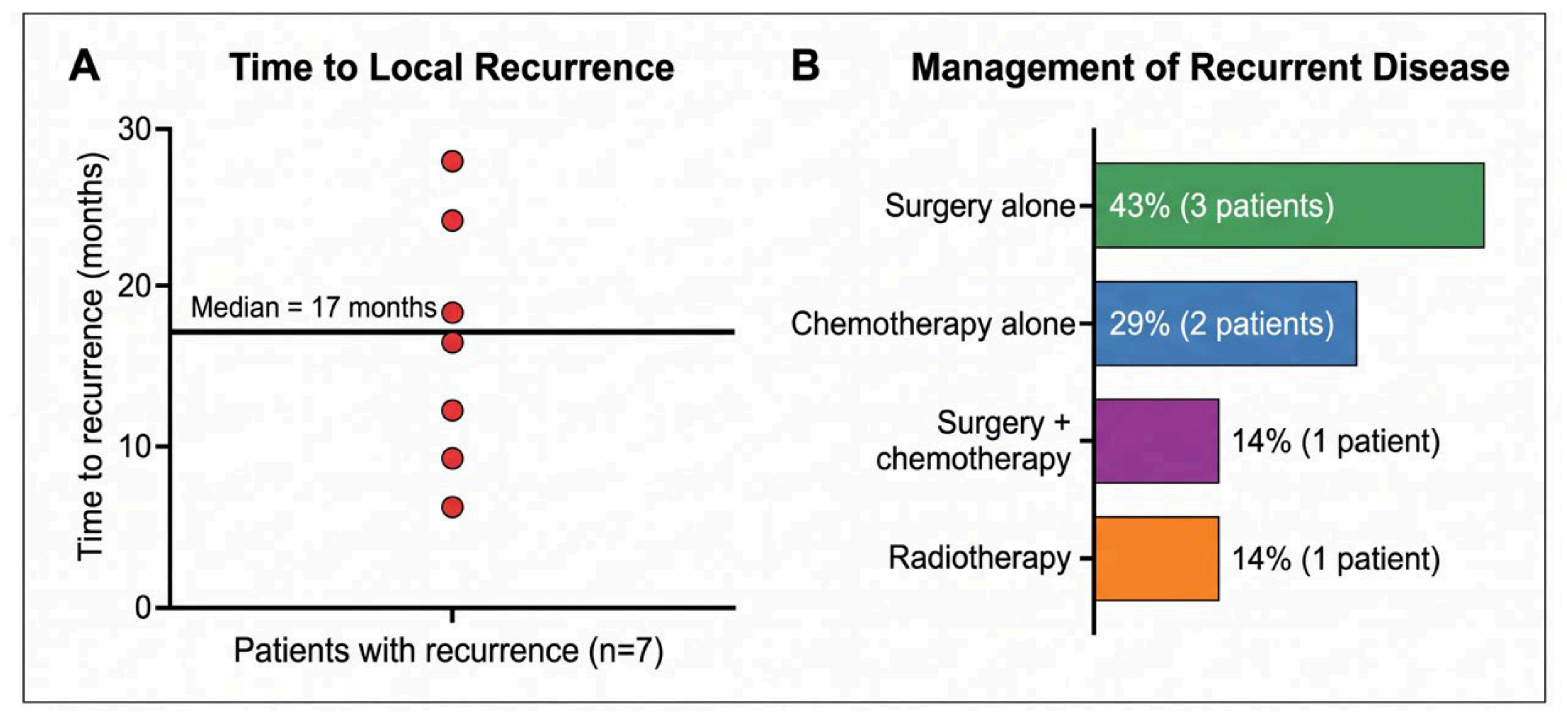
Recurrence outcomes. (**A**) Time to local recurrence (*n* = 7, 12.5% of cohort). Each circle = one patient. Bold horizontal line = median time to recurrence (17 months; range 6–28 months). (**B**) Management of recurrent disease (*n* = 7). Surgery alone: 42.9%; chemotherapy alone: 28.6%; surgery + chemotherapy: 14.3%; radiotherapy alone: 14.3%.

Local recurrence occurred in 7 of 56 patients (12.5%). The median time to recurrence was 17 months (range: 6–28 months) (Figure 3A). Management of recurrent disease included surgery alone (3/7, 42.9%), chemotherapy alone (2/7, 28.6%), combined surgery and chemotherapy (1/7, 14.3%), and radiotherapy (1/7, 14.3%) (Figure 3B).

In exploratory univariate Cox regression analysis (Table 4), positive lymph node status at diagnosis was the only variable associated with local recurrence (hazard ratio [HR] = 3.9, 95% confidence interval [CI]: 1.2–12.7, *p* = 0.02). Age, sex, HIV status, lesion morphology, and lesion count were not significantly associated with recurrence risk.

### 3.5. Metastasis

**Figure 4:**
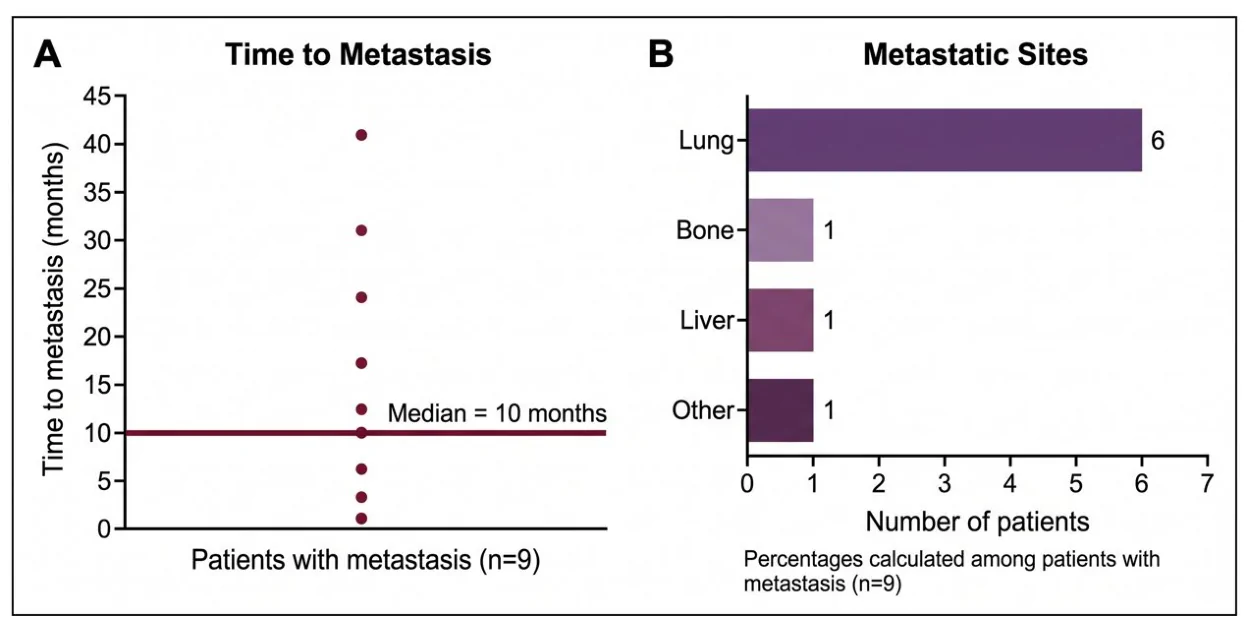
Metastasis outcomes. (**A**) Time to distant metastasis (*n* = 9, 16.1% of cohort). Each circle = one patient. Bold horizontal line = median time to metastasis (10 months; range 1–41 months). (**B**) Metastatic sites (*n* = 9). Lung: 66.7% (6/9); bone: 11.1% (1/9); liver: 11.1% (1/9); other: 11.1% (1/9).

Metastatic disease developed in 9 of 56 patients (16.1%), with a median time to metastasis of 10 months (range: 1–41 months) (Figure 4A). The lung was the most common metastatic site (6/9, 66.7%), followed by bone (1/9, 11.1%), liver (1/9, 11.1%), and other sites (1/9, 11.1%) (Figure 4B).

In exploratory multivariable Cox regression analysis (Table 5), adjusted for age, sex, and HIV status, two variables were associated with metastasis: nodular lesion morphology (adjusted HR = 4.8, 95% CI: 1.5–15.2, *p* = 0.007) and Ki-67 proliferation index ≥15% (adjusted HR = 5.2, 95% CI: 1.3–21.0, *p* = 0.02).

### 3.6. Overall Survival

At a median follow-up of 40 months (IQR: 16–70) among survivors, 9 deaths (16.1%) were recorded. Causes of death included KS progression (5/9, 55.6%), hepatocellular carcinoma (1/9, 11.1%), chronic kidney disease (1/9, 11.1%), and unknown causes (2/9, 22.2%).

**Figure 5:**
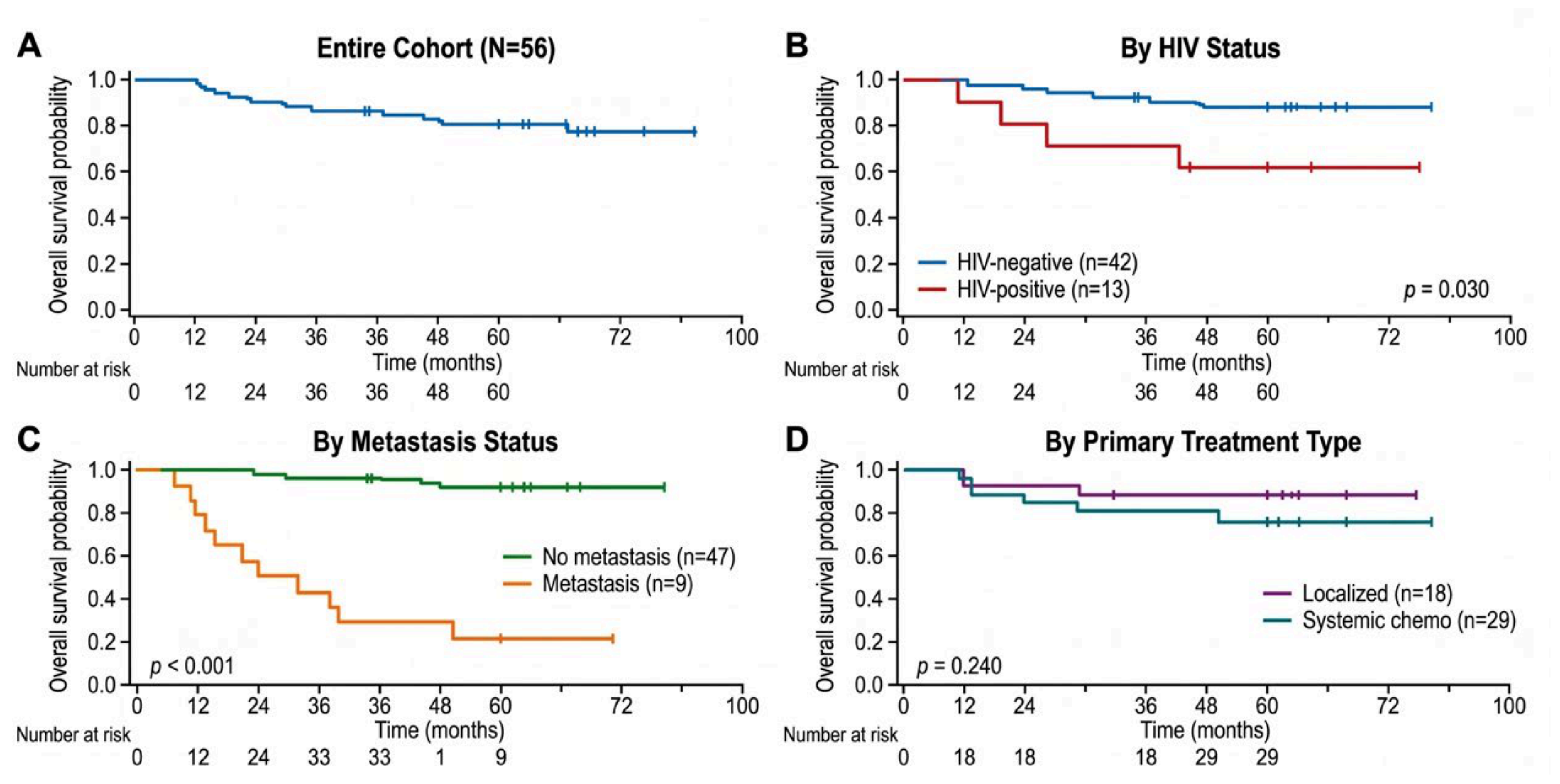
Overall survival analysis: Kaplan–Meier curves for overall survival (OS). Censoring ticks shown. Number at risk for each interval is provided in Table 6. (**A**) Entire cohort (N = 56). 1-, 3-, and 5-year OS: 94.6%, 85.7%, 80.4%. Median OS not reached (95% CI: 46 months—NE). (**B**) Stratified by HIV status (HIV-negative, *n* = 42; HIV-positive, *n* = 13; 1 patient with missing HIV status excluded). Log-rank *p* = 0.030. (**C**) Stratified by metastasis (*n* = 47 no metastasis, *n* = 9 metastasis). Log-rank *p* < 0.001. (**D**) Stratified by primary treatment (localized *n* = 18, systemic *n* = 29). Log-rank *p* = 0.240.

Kaplan–Meier estimates for overall survival (OS) are presented in Figure 5A and Table 6. Median OS was not reached (95% CI: 46 months—not estimable). The 1 year, 3 year, and 5 year OS rates were 94.6% (95% CI: 88.2–97.8%), 85.7% (95% CI: 76.4–91.7%), and 80.4% (95% CI: 69.5–87.9%), respectively.

On log-rank analysis, HIV-positive status was associated with significantly worse OS (median 46 months vs. not reached; log-rank *p* = 0.030) (Figure 5B). Development of metastasis also conferred poorer OS (median 33 months vs. not reached; log-rank *p* < 0.001) (Figure 5C). No significant difference in OS was observed between patients receiving primary systemic chemotherapy and those receiving localized treatment modalities (log-rank *p* = 0.240) (Figure 5D).

Multivariable Cox regression (Table 7), adjusted for age, sex, HIV status, metastasis, and nodular morphology, confirmed that HIV positivity (adjusted HR = 3.52, 95% CI: 1.14–10.87, *p* = 0.028) and development of metastasis (adjusted HR = 6.21, 95% CI: 1.98–19.48, *p* = 0.002) were associated with worse OS.

### 3.7. Disease-Free Survival

**Figure 6:**
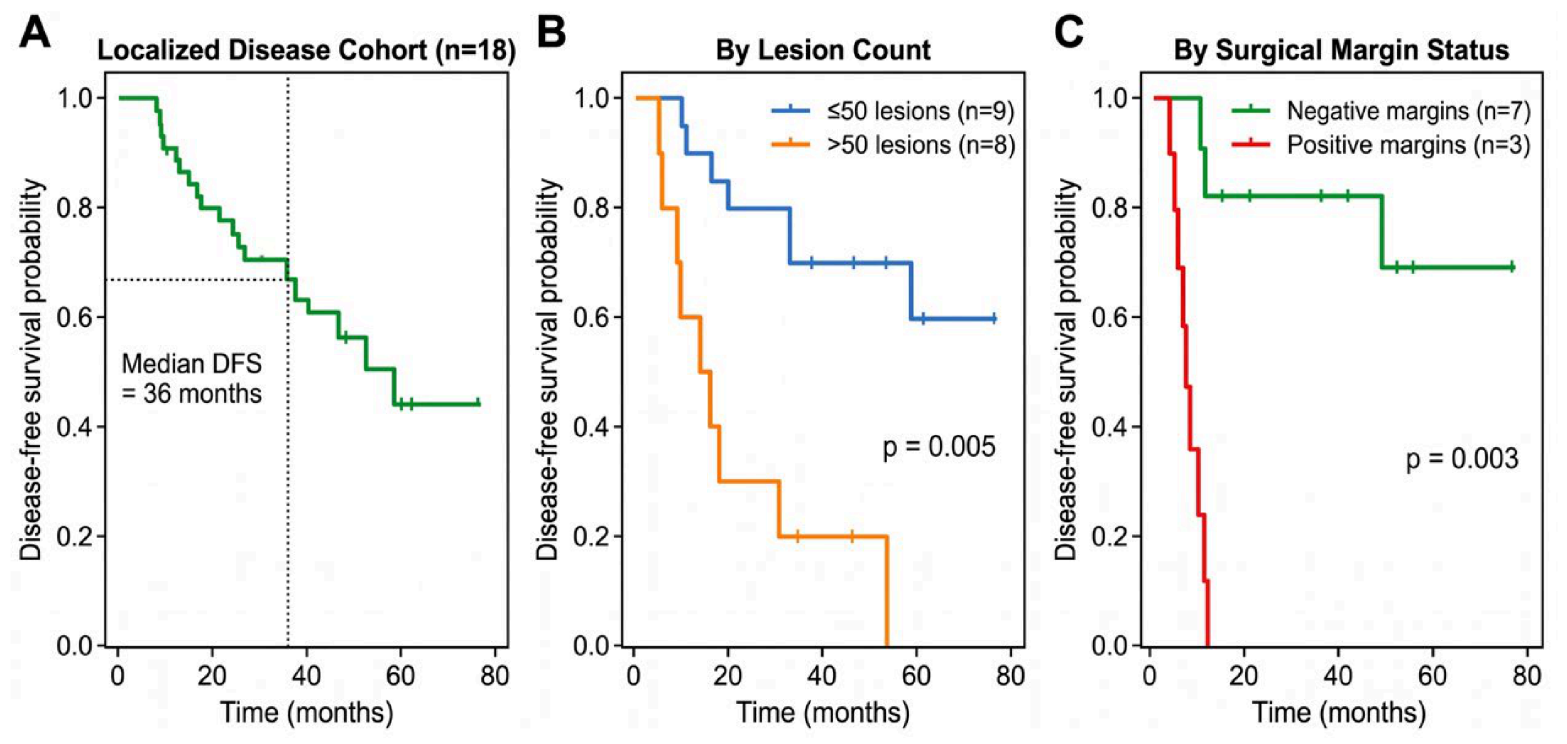
Disease-free survival among patients with localized disease (*n* = 18). Kaplan–Meier curves for disease-free survival (DFS). Censoring ticks shown. Number at risk for each interval is provided in Table 8. (**A**) Entire localized cohort. Median DFS = 36.0 months (95% CI: 17–60). 5-year DFS = 44.4%. (**B**) Stratified by lesion count (≤50 lesions, *n* = 9 vs. >50 lesions, *n* = 8). Log-rank *p* = 0.005. (**C**) Stratified by surgical margin status (negative *n* = 7, positive *n* = 3) among patients with evaluable margins (total *n* = 10). Log-rank *p* = 0.003.

Among patients with localized disease treated with curative intent (surgery, radiotherapy, or cryotherapy alone; *n* = 18), the median disease-free survival (DFS) was 36.0 months (95% CI: 17–60 months) (Figure 6A; Table 8). The 5-year DFS rate was 44.4% (95% CI: 27.4–62.3%).

Patients presenting with more than 50 lesions had significantly shorter DFS compared to those with ≤50 lesions (median 17 vs. 60 months; log-rank *p* = 0.005) (Figure 6B). Similarly, positive surgical margins (assessed in the 10 patients with available margin data) were associated with markedly worse DFS (median 9 vs. 48 months; log-rank *p* = 0.003) (Figure 6C).

### 3.8. Recurrence-Free and Metastasis-Free Survival

For the entire cohort, the 5-year recurrence-free survival (RFS) rate was 85.7% (95% CI: 76.2–91.9%), with a cumulative incidence of local recurrence of 14.3% (95% CI: 6.8–25.4%). The 5-year metastasis-free survival (MFS) rate was 82.1% (95% CI: 71.6–89.1%), with a cumulative incidence of metastasis of 17.9% (95% CI: 9.1–30.5%).

### 3.9. Subgroup Analysis

Subgroup analyses for overall survival are summarized in Table 9. HIV-negative patients had a 5-year OS rate of 88.1% compared to 61.5% among HIV-positive patients (log-rank *p* = 0.030). Patients who never developed metastasis had a 5-year OS rate of 91.5%, whereas those with metastasis had a 5-year OS rate of only 22.2% (*p* < 0.001). No significant difference in OS was observed by lesion count (≤50 vs. >50, *p* = 0.31), or lymph node status (negative vs. positive, *p* = 0.19).

### 3.10. Follow-Up

**Figure 7:**
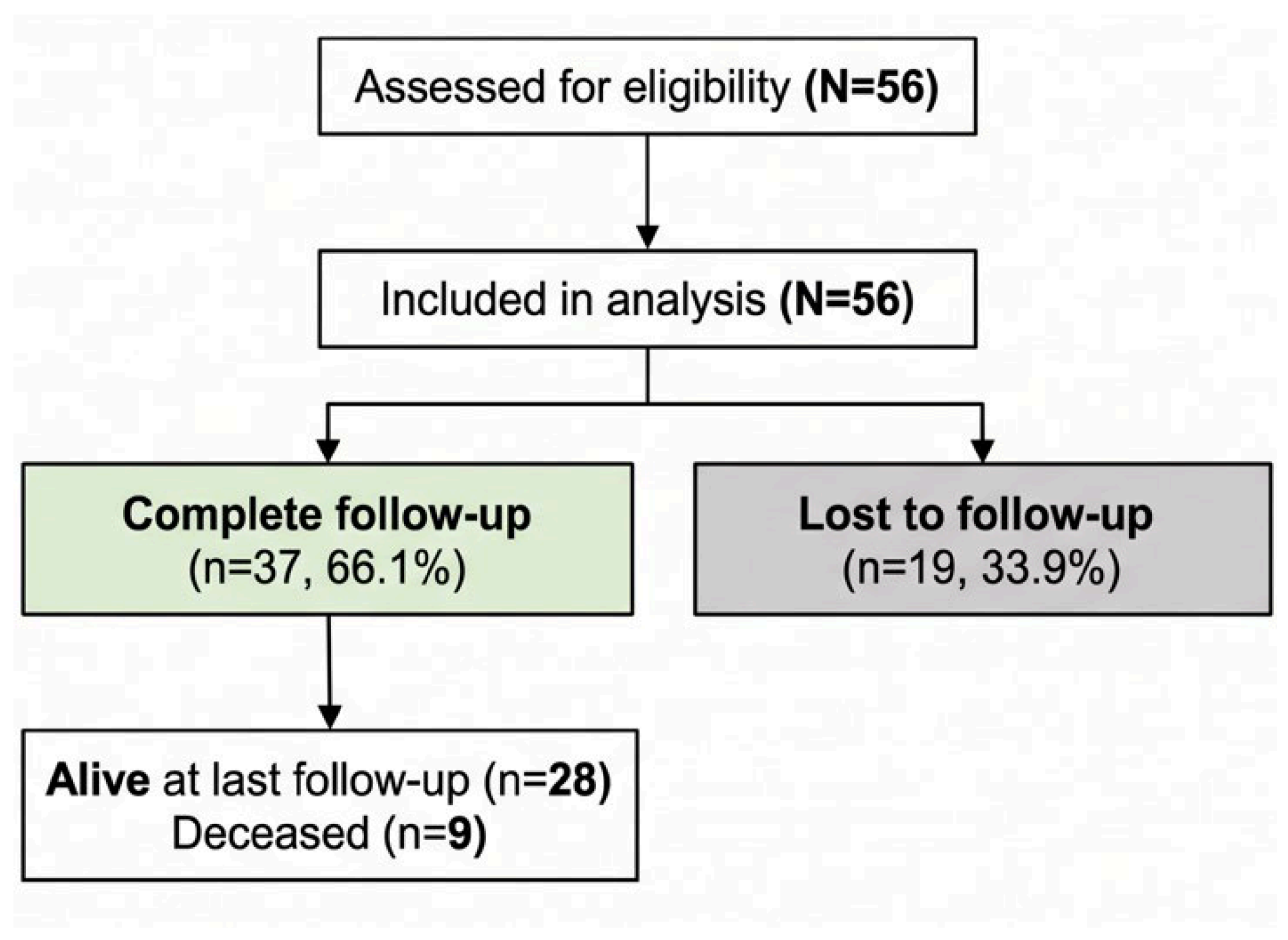
Patient disposition and follow-up status. Patient flow diagram. Total *N* = 56. Complete follow-up: 37 patients (66.1%; 28 alive, 9 dead). Lost to follow-up (no contact ≥12 months before data cutoff): 19 patients (33.9%).

Loss to follow-up occurred in 19 of 56 patients (33.9%) (Figure 7). No significant differences in baseline characteristics were observed between patients who completed follow-up and those lost to follow-up. However, the high rate of loss to follow-up may have biased survival estimates.

## 4. Discussion

This study presents an analysis of the clinical, pathological, and prognostic features of patients with KS. Our results show a median age of 61 years and a male predominance (82.1%), corresponding to that reported in the literature for the non-HIV types [8,9,10]. This highlights the importance of maintaining clinical suspicion in KS-mimicking lesions in older men, regardless of their HIV status.

**Figure 8:**
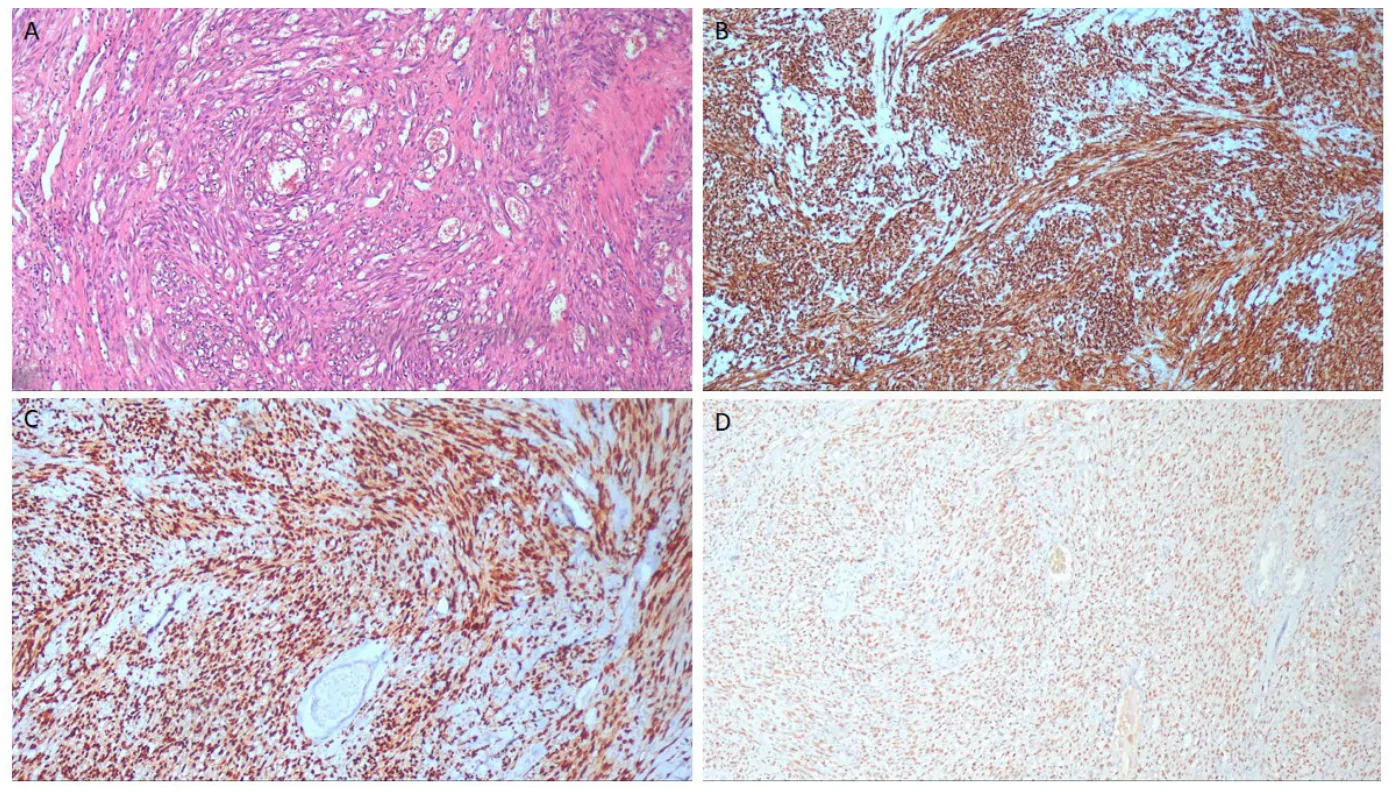
Microscopic examination of KS: Microscopic examination of a KS case of the presented cohort showing (**A**) Intersecting fascicles of spindle-shaped tumor cells with intervening sieve-like vascular spaces and extravasated RBCs. (**B**) CD34 immunostaining showing diffuse cytoplasmic positivity in tumor cells. (**C**) ERG immunostaining showing diffuse nuclear positivity in tumor cells. (**D**) HHV-8 immunostaining showing diffuse nuclear positivity in tumor cells.

The histopathological features of lesions (Figure 8) showed spindle cell proliferation in 96.0% of cases. They also confirmed the accuracy of the immunohistochemical (IHC) vascular markers. High positivity rates for HHV-8 (94.7%), CD34 (93.9%), and CD31 (95.5%) align with the literature, which defines these markers as gold-standard markers for KS diagnosis [11,12,13]. Our study also highlights the potential prognostic value of Ki-67, as high Ki-67 (≥15%) was associated with an increased risk of metastasis in exploratory analysis, supporting prior evidence that a high proliferation index is an indicator of KS aggressiveness [14,15]. However, these findings require validation in larger cohorts.

Analyzing the treatment approaches of our cohort showed that more than half of patients (51.8%) received systemic therapy, mainly paclitaxel (88.2%); a practice which is supported by previous studies demonstrating its efficacy as a first-line treatment for advanced or symptomatic KS [16,17,18]. For patients with localized disease, surgical excision and radiotherapy remained effective options. Our findings suggest that negative surgical margins were associated with significantly better disease-free survival (median 48 vs. 9 months). In exploratory analysis, negative surgical margins were associated with better disease-free survival; however, this finding is based on only 10 patients and should be interpreted with substantial caution.

Given the observational nature of the study, comparisons between systemic and localized treatment modalities are subject to confounding by indication. Patients receiving systemic therapy likely had more advanced or symptomatic disease compared to those receiving localized treatment. The absence of a significant difference in OS between treatment groups (log-rank *p* = 0.240) should therefore not be interpreted as evidence of treatment equivalence. These comparisons are exploratory and hypothesis-generating only.

A key finding of this study is that positive lymph node status was associated with local recurrence. Although KS is often viewed as a multicentric disease rather than a solitary tumor that spreads through lymphatics, regional lymph node involvement indicates a high tumor burden and a more aggressive behavior. However, this finding is limited by the small number of events (*n* = 7).

In exploratory multivariate analysis, nodular morphology and high Ki-67 were associated with distant metastasis. The lung was the most common metastatic site in our cohort, consistent with metastatic KS’s known tendency for lung spread [19]. The sharp decline in 5-year OS for patients with metastasis (22.2% vs. 91.5%) confirms the lethal nature of KS distant metastasis [20]. HIV-positive status and the development of metastasis were the main variables associated with worse OS. HIV-positive patients showed a significantly lower 5-year OS rate (61.5% vs. 88.1%), reflecting the importance of managing immunodeficiency as well as malignancy [21,22]. However, the association between HIV positivity and worse OS should be interpreted with caution due to the absence of data on CD4 count, HIV viral load, ART use, and ACTG stage.

Our study has limitations; firstly, being a retrospective study, because the disease is rare, heterogeneous, and often managed with individualized approaches. Selection bias, information bias, and unmeasured confounding are inherent limitations. Secondly, the small sample size and low event counts limit the generalizability of statistical analysis and lead to wide confidence intervals around all hazard ratios, reflecting imprecision and precluding definitive conclusions regarding independent prognostic factors. Thirdly, one-third of the patients were lost to follow-up, and missing data also introduced bias. Fourthly, the heterogeneity in treatment due to the long study period. Fifthly, competing risk analysis was not performed. Sixthly, no adjustment for multiple comparisons was performed in subgroup analyses. Seventhly, a major limitation of this study is the absence of key HIV-related variables, including CD4 count, HIV viral load, ART use, duration of HIV infection, and ACTG stage. Yet, the prospectively maintained database and the long median follow-up period, as well as the availability of main data such as the viral serostatus, helped present an addition to the already available literature.

## 5. Conclusions

While classic KS is a rare disease with an indolent behavior, exploratory analysis suggests that the presence of nodular morphology, high Ki-67, lymph node involvement, and HIV seropositivity is associated with worse outcomes. Systemic therapy remains the most frequently used treatment for advanced disease, while surgery and radiotherapy are appropriate options for selected localized lesions. These findings should be considered hypothesis-generating and require confirmation in larger, prospective, multicenter studies.

## Figures and Tables

**Table 1:** Baseline Demographic and Clinical Characteristics (*N* = 56).

Characteristic	*N* (%) or Median (Range)	Missing Data (*n*)
Age at diagnosis (years)	61 (27–89)	0
Sex		0
– Male	46 (82.1)	
– Female	10 (17.9)	
BMI (kg/m^2^) *	27.0 (16.0–47.6)	18
HIV status **		1
– Positive	13 (23.6)	
– Negative	42 (76.4)	
HCV status **		4
– Positive	11 (21.2)	
– Negative	41 (78.8)	
HBV status **		4
– Positive	1 (1.9)	
– Negative	51 (98.1)	

Abbreviations: BMI, body mass index; HIV, human immunodeficiency virus; HCV, hepatitis C virus; HBV, hepatitis B virus. * Among patients with available data (*n* = 38), ** percentages according to total number of patients with available data.

**Table 2:** Tumor and Pathological Characteristics.

Characteristic	*N* (%)	Missing Data (*n*)
Lesion morphology *		4
– Patch	9 (17.3)	
– Plaque	15 (28.8)	
– Nodular	28 (53.8)	
Number of lesions *		3
– <5	12 (22.6)	
– 5–50	16 (30.2)	
– >50	25 (47.2)	
Largest tumor diameter (cm) **	2.0 (0.5–30)	18
Lymph node status *		5
– Positive	21 (41.2)	
– Negative	30 (58.8)	
Spindle cells present *	48 (96.0)	6
Immunohistochemistry		
– HHV-8 positive	36/38 (94.7)	18
– CD34 positive	31/33 (93.9)	23
– CD31 positive	21/22 (95.5)	34
Ki-67 proliferation index		39
– High (≥15%)	7/17 (41.2)	
– Low (<15%)	10/17 (58.8)	

Abbreviations: HHV-8, human herpesvirus 8; CD34, cluster of differentiation 34; CD31, cluster of differentiation 31. * Percentages according to the total number of patients with available data, ** Median (range) among evaluable patients (*n* = 38).

**Table 3:** Primary Treatment Modalities.

Treatment Modality	*N* (%)
Systemic therapy	29 (51.8)
Surgical excision alone	13 (23.2)
Radiotherapy alone	3 (5.4)
Cryotherapy alone	2 (3.6)
Intralesional chemotherapy	2 (3.6)
Combined modalities	5 (8.9)
Unknown/missing	2 (3.6)

Systemic therapy as primary initial treatment: *n* = 29 (51.8%). Combined modalities: *n* = 5 (8.9%). Total patients ever receiving systemic therapy (including combined regimens): *n* = 34 (60.7%).

**Table 4:** Univariate Cox Regression Analysis for Local Recurrence.

Variable	HR	95% CI	*p*-Value
Age (per 10-year increase)	1.12	0.68–1.85	0.66
Male sex (vs. female)	1.38	0.30–6.34	0.68
HIV-positive (vs. negative)	1.82	0.41–8.09	0.44
HCV-positive (vs. negative)	1.45	0.32–6.57	0.63
Positive lymph node status (vs. negative)	3.92	1.21–12.70	0.02
Nodular morphology (vs. other)	2.12	0.48–9.36	0.32
>50 lesions (vs. ≤50)	1.88	0.41–8.62	0.42
Ki-67 ≥ 15% (vs. <15%)	2.34	0.52–10.53	0.27

Abbreviations: HR, hazard ratio; CI, confidence interval; HIV, human immunodeficiency virus; HCV, hepatitis C virus. Bold font indicates statistical significance (*p* < 0.05).

**Table 5:** Multivariable Cox regression Analysis for Metastasis.

Variable	Adjusted HR	95% CI	*p*-Value
Age (per 10-year increase)	1.15	0.68–1.94	0.60
Male sex (vs. female)	0.82	0.21–3.21	0.77
HIV-positive (vs. negative)	2.08	0.52–8.32	0.30
Nodular morphology (vs. other)	4.82	1.53–15.18	0.007
Ki-67 ≥ 15% (vs. <15%)	5.23	1.30–21.05	0.02
>50 lesions (vs. ≤50)	1.95	0.52–7.31	0.32

Abbreviations: HR, hazard ratio; CI, confidence interval; HIV, human immunodeficiency virus. Model adjusted for age, sex, and HIV status. Bold font indicates statistical significance (*p* < 0.05).

**Table 6:** Overall Survival—Kaplan–Meier Estimates (*N* = 56).

Time Point	Survival Probability (%)	95% CI	Number at Risk	Number of Events
12 months	94.6	88.2–97.8	48	3
24 months	89.3	81.0–94.2	41	5
36 months	85.7	76.4–91.7	35	6
48 months	82.1	72.0–89.0	28	7
60 months	80.4	69.5–87.9	22	7
72 months	78.6	67.2–86.7	16	8
84 months	78.6	67.2–86.7	10	8

Median overall survival: Not reached (95% CI: 46 months—not estimable) Mean overall survival: 68.4 months (95% CI: 58.2–78.6) Median follow-up among survivors: 40 months (IQR: 16–70). Abbreviation: CI, confidence interval; IQR, interquartile range.

**Table 7:** Multivariable Cox regression Analysis for Overall Survival.

Variable	Adjusted HR	95% CI	*p*-Value
Age (per 10-year increase)	1.18	0.75–1.86	0.47
Male sex (vs. female)	1.32	0.28–6.21	0.73
HIV-positive (vs. negative)	3.52	1.14–10.87	0.028
Metastasis (vs. no metastasis)	6.21	1.98–19.48	0.002
Nodular morphology (vs. other)	1.98	0.51–7.68	0.32

Abbreviations: HR, hazard ratio; CI, confidence interval; HIV, human immunodeficiency virus. Model adjusted for age, sex, HIV status, metastasis, and nodular morphology. Bold font indicates statistical significance (*p* < 0.05). Metastasis was included as a time-fixed covariate, which is subject to immortal time bias. Kaplan–Meier estimates should be interpreted with caution as competing risk analysis was not performed.

**Table 8:** Disease-Free Survival—Kaplan–Meier Estimates (Localized Disease, *n* = 18).

Time Point	DFS Probability (%)	95% CI	Number at Risk	Number of Events
12 months	88.9	76.2–96.3	16	2
24 months	77.8	62.5–89.0	13	3
36 months	66.7	49.6–80.6	10	4
48 months	55.6	38.1–71.7	7	5
60 months	44.4	27.4–62.3	4	6

Median disease-free survival: 36.0 months (95% CI: 17–60). Mean disease-free survival: 42.3 months (95% CI: 30.5–54.1) Abbreviations: DFS, disease-free survival; CI, confidence interval. Localized disease is defined as patients treated with surgery, radiotherapy, or cryotherapy alone.

**Table 9:** Subgroup Analysis—Overall Survival by Key Clinical Variables.

Subgroup	*N*	Events	Median OS (Months)			95% CI	5-Year OS (%)	Log-Rank *p*
HIV status								
– HIV-negative	42	5	NR			46–NR	88.1	
– HIV-positive	13	4	46.0			33–56	61.5	*p* = 0.030
Metastasis								
– No metastasis	47	4	NR	48–NR	91.5			
– Metastasis	9	5	33.0	20–46	22.2	*p* < 0.001		
Primary treatment								
Lesion count								
– ≤50 lesions	28	3	NR	48–NR	89.3			
– >50 lesions	25	5	NR	42–NR	76.0	*p* = 0.31		
Lymph node status								
– Negative	30	3	NR	48–NR	90.0			
– Positive	21	5	NR	40–NR	71.4	*p* = 0.19		

Abbreviations: OS, overall survival; NR, not reached; CI, confidence interval; HIV, human immunodeficiency virus.

## Data Availability

All the clinical, radiological and pathological data used in this manuscript are available in the Mansoura University medical system (Ibn Sina Hospital management system). The R scripts used to generate Figure 4, Figure 6 and Figure 7 are available from the corresponding author.

## Abbreviations

ACTG	AIDS Clinical Trials Group
AIDS	Acquired Immunodeficiency Syndrome
BMI	Body Mass Index
cART	Combination Antiretroviral Therapy
CD31	Cluster of Differentiation 31
CD34	Cluster of Differentiation 34
CI	Confidence Interval
DFS	Disease-Free Survival
GLOBOCAN	Global Cancer Observatory
HBV	Hepatitis B Virus
HBVsAg	Hepatitis B Virus Surface Antigen
HCV	Hepatitis C Virus
HCVab	Hepatitis C Virus Antibody
HHV-8	Human Herpesvirus 8
HIV	Human Immunodeficiency Virus
HIVab	Human Immunodeficiency Virus Antibody
HR	Hazard Ratio
IHC	Immunohistochemistry
IQR	Interquartile Range
IRB	Institutional Review Board
KS	Kaposi Sarcoma
MFS	Metastasis-Free Survival
OS	Overall Survival
RFS	Recurrence-Free Survival
